# How Does Understanding of Social Situations and Other’s Intention Contribute to Idiom and Irony Comprehension in Autism Spectrum Disorder?

**DOI:** 10.3390/brainsci14101034

**Published:** 2024-10-18

**Authors:** Nira Mashal, Naama Lellouche

**Affiliations:** The Faculty of Education, Bar-Ilan University, Ramat Gan 5290002, Israel; naama.lellouche@gmail.com

**Keywords:** ASD, irony, idioms, pragmatics, ToM, ironic criticism, social cognition

## Abstract

**Background/Objectives:** Figurative language is a central tool for enriching spoken and written languages, and it is important for building social relationships. Difficulties in figurative language understanding may impair social adjustment. Some studies have found more gaps in the understanding of irony and idioms among children with autism spectrum disorder (ASD) compared to those of their peers with typical development (TD). To date, no studies have examined the relationship between the ability to understand social situations (as a separate ability) and the ability to understand irony and idioms. The present study examined the roles of theory of mind (ToM) and social situation understanding in the comprehension of idioms and ironic criticism. **Methods**: The current study included 58 participants aged 8–11, including 28 children with high-functioning ASD and 30 children with TD matched by age, gender, and nonverbal intelligence. All the participants completed a ToM questionnaire that assesses their understanding of others’ intentions, as well as a questionnaire pertaining to their comprehension of social situations, ironic criticism, and idioms. **Results**: TD children outperformed the autistic children in idiom and irony understanding, as well as in ToM and social situation understanding. Understanding social situations and ToM contributed to idiom and irony understanding, with ToM ability uniquely contributing to irony (but not to idiom) understanding. Path analysis revealed that social cognitive abilities mediated the link between group affiliation and vocabulary, affecting figurative language comprehension. **Conclusions**: The present study demonstrates that social cognition skills influence the ability to understand ironic criticism and idioms, mediating the association between vocabulary and figurative language comprehension.

## 1. Introduction

The ability to use and understand language and other expressive means in a social context refers to pragmatics [1]. Pragmatics is a broad domain encompassing figurative language that serves as a central tool for communicating non-literal meanings, i.e., meanings that do not correspond to the word-by-word interpretations. The different aspects of figurative language, including, for example, metaphors, humor, irony, and idioms, are characterized by a gap between the literal meaning of the figurative expression and the message the speaker intends to convey. Figurative language contravenes Grice’s maxim of quality, which states that the speaker should convey to the listener precise, true, and verifiable information [2]. Understanding all types of figurative language is thus dependent on pragmatic abilities, or in other words, on the listener’s ability to go beyond the word-by-word comprehension strategy, retrieve the figurative interpretation from their mental lexicon, or compute the expression’s meaning by inferential processes to fill the gap between what was literally said and what was meant [3,4,5,6]. Consequently, difficulties in figurative language understanding may negatively affect social interactions and social adjustment [7].

Two common types of figurative language are idioms and irony. Idioms are considerably fixed, lexicalized figurative phrases that, according to the Global Elaboration Hypothesis [8], develop along with users’ general linguistic and cognitive development. Idioms vary in several dimensions, such as the level of familiarity, transparency (the extent to which the meaning of the individual words contributes to the figurative meaning), and literal plausibility (the extent to which the literal meaning is plausible). Other studies have shown that these dimensions affect listeners’ ability to understand idioms throughout life [9]. For example, 5-year-old children find it easier to understand transparent idioms than opaque ones [10]. Nine-year-old children can rely on the transparency of an idiom to understand its meaning out of context, but seven-year-olds cannot [8]. Fourteen-year-old adolescents outperformed eleven-year-old children in explaining the meaning of transparent idioms [11], attesting to the contribution of age to the development of idiom understanding [12].

There are several subtypes of verbal irony (ironic criticism, ironic compliments, hyperboles, and understatements), but in the current study, we adopt the definition of irony as a statement in which a speaker intentionally communicates insincerely, aiming for the listener to perceive the contradiction between the situation and the words presented [13], focusing on ironic criticism. Specifically, we use a form of irony that is represented by a literally positive remark that conveys criticism. For example, Romi’s neighbors are renovating their home, and the workers are drilling non-stop. When the neighbors asked Romi about the noise, Romi replied: “It’s as quiet as a library”. This form of irony differs from ironic compliments (not used in the current study), in which the speaker uses a negative remark to convey a compliment. The latter is more difficult to understand than ironic criticism probably because ironic compliments require the negation of a negative statement [14,15]. It has been suggested that the interplay between literal meanings and contextually relevant information is crucial for grasping the speaker’s intended implicit message [16]. Therefore, the ironic statement becomes more complex to decipher, and possibly leads to misunderstandings and difficulties in social communication.

Although some studies have shown that irony understanding begins to develop around the age 5–6 [17,18,19], there is evidence suggesting that children begin to show initial signs of irony comprehension at ages 3 and 4 years old [20,21]. Children continue to develop this ability through middle childhood, at approximately 7–10 years old [22]. Nonetheless, the evidence suggests that irony comprehension continues to develop into adolescence [23,24], with 9-year-old children understanding irony less than adults [25]. Thus, whereas idioms are lexicalized items stored in the mental lexicon, the utilization of irony requires the listener to replace the literal meaning expressed in the statement with the intended or implied meaning. Understanding the implied meaning of an ironic statement may demand blending the literal meaning with the social setting of the communication, therefore requiring social cognition competence. The current study seeks to focus on children aged 8–11 years old, an age range in which the understanding of figurative language is still developing and has not yet reached its peak [26].

Autism spectrum disorder (ASD) is a neurodevelopmental condition marked by difficulties in communication and social interactions. The inability to grasp nonliteral meanings is included in the DSM-5 diagnostic criteria for ASD [27]. Research has shown that children with autism often interpret conversations literally and struggle to recognize the speaker’s true intent, contributing to challenges in social communication and interaction [15]. As a result, many researchers have begun investigating the ability of individuals with ASD to understand figurative language based on studies highlighting their difficulties with nonliteral language comprehension.

Pragmatics is widely recognized as the most consistently and universally affected linguistic domain in autism [28], even among individuals who exhibit typical structural language abilities. Within the scope of pragmatics, individuals with autism often struggle with interpreting others’ communicative intentions, a key skill needed for understanding figurative language. Research indicates that individuals with autism have a reduced comprehension of figurative expressions, such as metaphors, idioms, and irony [15,29,30,31,32,33,34,35], compared to that of typically developing (TD) individuals, with a tendency toward literal interpretations [31,36]. A diminished understanding of idioms and humor has also been observed in adolescents with ASD aged 12–15 compared to that of their TD peers matched for age, gender, and vocabulary knowledge [37]. Several theories and models have been proposed to explain these difficulties in figurative language comprehension in individuals with ASD. One prominent theory, the theory of mind (ToM) [38,39], suggests that people with ASD struggle to understand the mental states of others. Consequently, they may have difficulty grasping social situations and communication aimed at them.

The term “theory of mind” was originally introduced to describe the ability to attribute mental states to oneself and others. However, the false-belief task [28], which measures the understanding of mistaken beliefs, quickly became the primary test for assessing this ability in children [40]. This narrow focus led to an emphasis on studying 3- to 5-year-olds, the age range when children typically succeed in false-belief tasks, and on mental states like belief and knowledge rather than other aspects like intentions or emotions. Although false-belief comprehension is just one indicator of a broader developmental capacity, it is linked to key social skills, including pretend play, communication, and handling criticism. Despite these findings, there is a paradox: 3-year-olds often fail false-belief tasks, yet show competence in daily social interactions, raising questions about whether this definition of “theory of mind” truly captures children’s social abilities [41]. Consequently, many researchers now adopt broader definitions of “theory of mind”, incorporating a wider range of mental states, such as perception, intention, cognition, and emotion, which significantly expands the field of study [42]. Importantly, research indicates that advancements in ToM predict not only children’s social skills and interactions, but also their cognitive abilities and behaviors [40]. Accordingly, it has been suggested that advances in ToM not only reshape children’s close relationships, but also underscore the complexity of these socio-cognitive influences and the need for further research in this area [41].

Other studies on ToM abilities have expanded to also include the understanding of the link between ToM and pragmatics and figurative language in particular. ToM ability has been shown to predict figurative language processing in both children and adults with ASD [39]. The evidence suggests that this association between ToM abilities and the ability to understand idioms is evident among children with ASD, but not among children with TD [43]. Furthermore, it has been suggested that first-order ToM ability is sufficient for understanding metaphors, but not for understanding irony, whereas second-order ToM ability contributes to the understanding of metaphors and irony [39]. A recent study refined this association (between irony and ToM ability) and found links between second-order ToM and ironic compliments and between linguistic abilities and ironic criticisms in children with ASD (mean age = 7.3 years) [15]. The role of ToM ability in irony comprehension has also been examined in recent studies [12,44,45], in which participants with TD outperformed the group with ASD in irony comprehension. It is noteworthy that when the participants with ASD were matched for the ability to understand other’s intention using the Hinting test [12], both the groups exhibited a similar performance in the irony comprehension task. Furthermore, second-order ToM ability correlated with irony comprehension and mediated the link between the severity of ASD symptoms and irony comprehension [45]. Thus, most of the studies point to a link between ToM ability and the understanding of idioms and irony among individuals with ASD.

Other researchers attribute challenges individuals with autism experience in understanding figurative language to the difficulty they experience in performing executive functions. Limited mental flexibility or working memory can impair the ability to inhibit irrelevant interpretation or to shift between the literal and the non-literal interpretations of a figurative expression [46,47]. Only a few studies tested the correlation between figurative language and executive functioning in ASD. When the relationship was examined, no relationship was found between irony comprehension and different components of executive functions, including spatial working, memory tasks, inhibitory control, and cognitive flexibility [45,47]. Thus, the link between executive functions and idiom and irony comprehension remains largely unknown.

Another approach to explaining the difficulties in understanding figurative language among individuals with ASD is based on acknowledging their general difficulty in understanding language [48]. Supporting this approach, a recent meta-analysis [29] found that when the participants were matched according to their language abilities, and especially according to vocabulary and syntactic capabilities, no significant differences were observed in figurative language understanding between the groups with ASD and with TD. The unique contribution of vocabulary to idiom comprehension performance among individuals with ASD was demonstrated in a recent study that showed that vocabulary contributed significantly to idiom comprehension performance, beyond the contributions of age and gender among the participants with ASD, but did not do so among their TD peers [12].

Social cognition encompasses a range of subdomains, but at its core, it involves the mental processes involved in social interactions. This includes how we perceive and understand others’ intentions, personalities, and actions (i.e., ToM abilities), as well as how we formulate responses to these interactions [49]. In other words, social cognition encompasses all cognitive processes directed towards recognizing and interpreting information obtained from social contexts, comprehending one’s own and/or others’ behavior, and adjusting thought and action patterns according to the demands of varying social scenarios [50]. In the current study, we focus on two aspects of social cognition: understanding other’s intentions (as assessed by the Hinting test) and understanding social situations (as assessed by the Children’s Social Comprehension Scale, CSCS). Both these tests are often used in research and clinical settings to evaluate theory of mind deficits in conditions such as ASD and schizophrenia. In the Hinting task, participants are typically presented with short scenarios involving social interactions where a character drops hints about their intentions or beliefs. The participants are then asked to infer what the character is trying to communicate. The CSCS is designed to evaluate a child’s understanding of problematic social situations using social cues. It assesses various aspects of social situation comprehension, including interpretation of social scenarios and the understanding of the consequences of violating social norms. Children are asked to decide what the worst thing is in that situation. Its relationship with figurative language understanding in children with autism has not been tested before.

There is a growing interest in studying the association between social cognition and neurodevelopmental disorders (for review [5,51]), yet limited research has delved into this aspect, while considering language, and in particular, figurative language proficiency. For instance, Im-Bolter et al. [7] studied adolescents referred to a mental health center (clinical group) as previous studies indicated that these adolescents show decreased figurative language comprehension as compared to controls (non-clinical group [52]). Social cognition was measured using the Interpersonal Negotiation Strategies interview [53] that assesses social cognitive maturity. The findings indicated that age, working memory, and both structural and figurative language skills are linked to social cognitive maturity in the clinical group, with only structural language linked to social cognitive maturity in the non-clinical group. This interaction between social cognition and figurative language skills holds significant implications in ASD as children with autism often exhibit deficits in both ToM abilities and various aspects of pragmatics (in particular, figurative language) proficiency. Yet, there are only a few studies that simultaneously assessed how both the structural and pragmatic aspects of language contribute to understanding the extent of social impairments in children with autism. It has been suggested that while pragmatic challenges have a notable correlation with heightened social impairments in children aged 7–17 with ASD, structural language difficulties do not show a significant association [54].

The overarching goal of the present study is to examine the relationship between the ability to understand social situations and other’s intention and the ability to understand figurative language. As adolescents rapidly develop the ability to comprehend figurative language, which becomes increasingly prevalent in peer interactions [55], it is imperative to fill the gap regarding the link between figurative language and social cognition in ASD. Using a questionnaire focused on the understanding of other’s intention (the Hinting test) and a questionnaire for understanding social situations (CSCS), the current study seeks to assess, for the first time, the association between these social cognitive skills and idiom and irony comprehension among children with and without ASD. The aims of the present study are thus threefold: (1) to examine the understanding of idioms, irony, and social situations in children with ASD compared to children with TD; (2) to examine what abilities contribute to the understanding of irony and idioms, with a specific focus on the contributions of vocabulary, understanding other’s intention, and social situation comprehension; and (3) to determine what is the roll of the social cognitive skills in the relationship between group affiliation and figurative language understanding. We hypothesized that the children with TD would outperform the ASD group in terms of their comprehension of idioms, irony, and social situations [12,28,33,34,45,56]. We also hypothesized that vocabulary and the understanding of both social situations and others’ intentions would contribute to the explained variance in irony understanding. Unlike idiomatic expressions, ironic expressions depend more heavily on understanding the social context and the speaker’s intention, and on more advanced social-cognitive reasoning [25]. We thus presumed that vocabulary [12], understanding social situations, and other’s intention all contribute only to irony comprehension [45,57]. Finally, we hypothesized that social cognitive skills (ToM and social situation understanding) mediate the link between group affiliation and figurative language understanding (idiom and irony comprehension).

## 2. Materials and Methods

### 2.1. Participants

In total, 58 participants aged 8–11 in grades 3–6, including 28 children with a formal diagnosis of ASD, participated in the study. The participants with ASD were diagnosed by psychologists or psychiatrists according to the DSM-5 [27] criteria. The clinical diagnosis of these participants was confirmed using the Social Communication Questionnaire (SCQ). The parents of children with ASD answered the SCQ [58], which is a parental report questionnaire designed to determine whether their child falls within the autism spectrum. The SCQ included 40 items related to the areas of communication, mutual social communication, interests, and repetitive and stereotypical activities. A score above 15 confirmed the diagnosis of ASD. The SCQ was scored by an MA student specialized in ASD. All the participants in the ASD group scored 15 and above on this questionnaire. The participants were recruited from communication classes in a mainstream education school in the south of the country. The participants in the control group exhibited typical development, with no history of neurodevelopmental disorders or psychiatric diagnoses according to parents’ reports. Control participants were recruited through relatives, acquaintances, and friends. They were matched groupwise to the ASD group based on their age, gender and nonverbal cognitive ability. The participants and their parents provided written informed consent before testing. Table 1 shows the background characteristics of both the groups.

As shown in Table 1, no significant difference was found between the groups in terms of age, gender, or nonverbal intelligence. However, the children with TD scored higher on vocabulary than did their ASD peers.

### 2.2. Materials

#### 2.2.1. Verbal and Nonverbal Intelligence Tests

Vocabulary was assessed using the vocabulary subtest from the Wechsler Intelligent Scale for children (Wechsler Intelligent Scale, WISC-IVHEB) [59]. The participants were asked to provide definitions of words they heard.

Nonverbal intelligence was assessed using the RAVEN test (CPM Raven’s Colored Progressive Matrices) [60]. The test included 36 items divided into 3 sets, with 12 items in each set. The items were arranged in increasing difficulty order, as were the three sets in the test. For each item, the subject had to choose the missing part that completed the picture shown to them. There was one correct answer out of six options. Correct and incorrect answers received scores of 1 and 0, respectively, with a maximum score of 36.

#### 2.2.2. Social Cognitive Assessment

The Hinting test [61] evaluates the understanding of the other’s intentions (ToM ability) and has been tested in patients with schizophrenia and children and adolescents with ASD [12,62]. The participants were presented with 10 short stories describing a situation involving two characters. At the end of each story, a question was posed related to the understanding of the speaker’s intention, which was not explicitly stated in the story. For example: “Karen’s birthday is coming up. Karen says to her father, ‘I love animals, especially dogs’. Question: ‘What does Karen really mean when she says that?’”. If the subject answered incorrectly, a hint is provided: “Dad, will the pet store be open on my birthday?”. A correct answer was awarded two points. The maximum score for this test was 20 points. If the participant initially answered incorrectly, but was able to answer correctly using the hint, 1 point was awarded.

**Figure 1:**
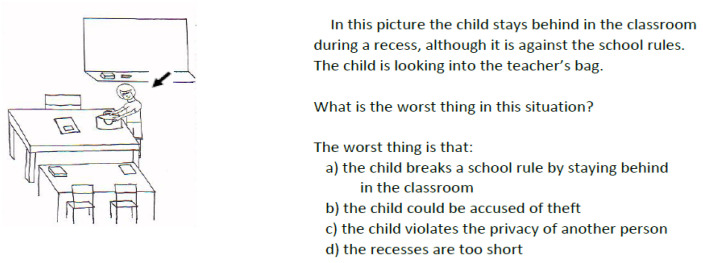
Example from the Children’s Social Comprehension Scale.

The Children’s Social Comprehension Scale (CSCS) [63] is a social comprehension scale questionnaire for children aged 6–11-years-old. The questionnaire assesses the ability to encode social information as well as the respondent’s understanding and interpretation of human behavior in social situations. The questionnaire is used to measure the cognitive component of social intelligence among young school-aged children (Figure 1), and it requires knowledge regarding social norms, the principles behind them, and the consequences of violating these norms [63].

The questionnaire in this study included 10 items consisting of short stories accompanied by pictures (see Figure 1). The stories described diverse social situations, such as gossiping, bullying, violating privacy laws, and not sharing with a friend. The participant was asked to identify what was the worst thing in the specific situation. The question presented at the end of each story was a multiple choice question with four possible answers, one of which was correct. The participants scored one point for a correct answer and zero points for a wrong answer. The maximum possible score was 10 points. Cronbach’s alpha test reliability coefficient values for the CSCS are 0.68 for ages 6–7, 0.75 for ages 8–9, and 0.89 for ages 10–11. The validity of the test, according to the confirmatory fit index, is above 0.95 [63].

#### 2.2.3. Figurative Language Questionnaires

The idiom questionnaire [31] tests the ability to understand idioms. This questionnaire is a multiple choice test that consists of 20 idioms. In this study, for each idiom, four choices were presented: (1) the correct answer; (2) an incorrect literal answer; (3) another literal distractor; and (4) an unrelated answer. The proposed options were displayed in a random order. The participants were required to select the answer closest to the meaning of the entire sentence. For example, for the idiom “Rubbing salt on the wounds”, four alternatives were presented: (1) spice spreader; (2) disinfecting the warts; (3) talking about other people’s failures and thereby causing them additional pain; and (4) listening to others. The participants received one point for each correct answer.

The irony comprehension questionnaire [34] included 15 items, of which 10 included short text passages with ironic meaning and the remaining 5 included short passages with literal meaning. The sections in the questionnaire were presented in a random order. The participants were asked to read each passage and answer an open-ended question that referred to the intention or thought of the speaker. For example, “The final exam lasted for about three hours, covered a lot of material, and included material that was not studied at all. At the end of the test, the students said to the teacher: ‘The test was easy’. What did the students think about the test?”. The participants received one point for a correct answer, with a maximum of 10 points for the ironic portion and 5 points for the literal portion.

### 2.3. Procedure

The participants and their parents signed a consent form that was approved by the chief scientist of the Ministry of Education and the ethics committee of the university. The parents were provided with an explanation of the purpose of the study and the manner of its execution. At the beginning of the meeting, the participants received a general explanation of the study and answered the questionnaires individually. The questionnaires were delivered orally by the researcher, who recorded the responses in written form, except the SCQ that was delivered electronically to the parents of the participants with ASD using Google Forms. Each participant completed the tests in a quiet room in the participant’s home or at the school during one session that lasted 60–90 min, including a break as needed. The tests were administered in a random order to exclude possible effects between the tests.

### 2.4. Data Analysis

Prior to examining the differences between the children with TD and ASD in idioms, irony, ToM and social situation understanding, Shapiro–Wilk analysis was conducted for each study measure to determine whether the distribution of these measures was normally distributed and to test whether the assumptions for conducting one-way MANCOVA analysis had not been violated. The results indicated that the distributions of most study measures significantly deviated from normality (*p* < 0.05). Therefore, to investigate the differences between the children with TD and ASD in idioms, irony, ToM, and social understanding, we used partial correlations to examine the associations between these variables and group, while controlling vocabulary. To test to what extent understanding social situations and ToM abilities contribute to understanding irony or idioms (as dependent variables), hierarchical regression analysis was performed for each group separately. For these statistical analyses, the SPSS software package version 20.0 was used. Finally, path analysis was conducted to capture the relationships between vocabulary, group affiliation, ToM, social situation understanding, and both idiom and irony comprehension using AMOS v. 29.

## 3. Results

### 3.1. Comparing Idiom, Irony, ToM, and Social Understanding Between Groups

The results of partial correlation analysis indicated a significant association between idioms, irony, ToM (as assessed by Hinting), and understanding social (as assessed by CSCS) and group situations, with vocabulary treated as a covariate [*r* = 0.33, *p* = 0.012, *r* = 0.57, *p* < 0.001, *r* = 0.65, *p* < 0.001 and *r* = 0.47, *p* < 0.001, respectively]. The positive correlations indicate that the TD children outperformed the children with autism.

### 3.2. The Contributions of Demographic and Background Variables, ToM, Understanding Social Situations, and Group Affiliation to Idioms and Irony Comprehension

Two hierarchical regression models were constructed, with one being used to predict irony comprehension, while the other was used to predict idiom comprehension. In each model, in the first block, age and gender were entered as controlled variables. In the second block, vocabulary as well as nonverbal intelligence were entered. In the third block, group affiliation, the understanding of social situations, and ToM (centered) were entered. In the fourth block, the interaction factors between the groups and the understanding of social situations and ToM were entered (Table 2).

As shown in Table 2, the first block was not significant in either model, with 1.9% and 4.8% explained variance (EPV) for the idiom and irony models, respectively. The second block, in which the vocabulary and nonverbal intelligence were entered, was significant for both the models, with increases in EPV of 81.0% and 57.3% for the idiom and irony models, respectively. Further examination of the coefficients revealed that for both the models, only vocabulary had a significant unique contribution, with a higher level of verbal intelligence predicting a better understanding of idioms and irony. In the third block, group affiliation, understanding social situations, and ToM were found to contribute significantly to both the models, with 2.8% and 32.1% increases in EPV, respectively. An examination of the associated coefficients revealed that none of the variables significantly predicted idiom comprehension, although the entire block was significant. In the irony understanding model, a significant unique contribution was found for both the group affiliation and ToM. The fourth block did not yield any significant contributions for the idiom or irony models, with 0.00% and 0.2% respective increases in EPV. This result indicates that the relationships found in the third block did not differ between the two groups.

### 3.3. Path Analysis

Finally, path analysis was conducted to reduce multicollinearity between the explanatory variables (such as group and vocabulary) and to enhance clarity, while accounting for the correlation between the two dependent variables: idiom and irony comprehension. Composite (average) scores were constructed for the social cognitive skills (combining Hinting and CSCS tasks) and for figurative language (irony and idiom comprehension tasks). This approach is consistent with the recommendations for reducing complexity, as it minimizes multicollinearity and yields more reliable results. Furthermore, using composite scores simplifies analysis and allows us to conduct path analysis with smaller required sample sizes [64].

Given the limited sample size in the current study, we conducted path analysis instead of structural equation modeling (SEM). The CMIN and RMSEA indices are particularly sensitive to sample size; in our analyses, these values indicated an inadequate goodness of fit [65]. In this study, we examined the model using several goodness-of-fit indices: the χ^2^/df ratio (CMIN), the Comparative Fit Index (CFI), the Tucker–Lewis Index (TLI), the Incremental Fit Index (IFI), and the Root Mean Square Error of Approximation (RMSEA). According to Hu and Bentler (1999) [65] and Hair et al. (2006) [66], a very good fit is defined as a relatively small chi-square ratio (χ^2^/df ≤ 3), with a CFI, a TLI, and an IFI ≥ 0.95, and an RMSEA ≤ 0.06. An adequate fit to the data is indicated by a CFI, a TLI, and an IFI greater than 0.90 and an SRMR lower than 0.08.

**Figure 2:**
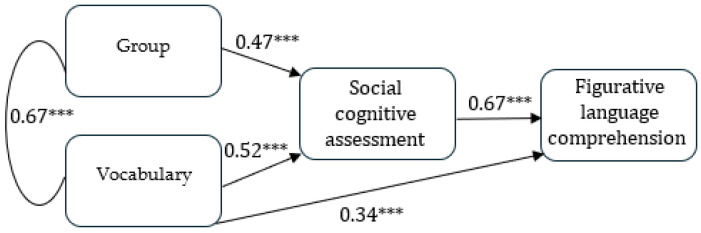
Path analysis. *** = *p* < 0.001.

The results of the model yielded a very good fit of the indices: CMIN = 1.322, CFI = 0.999, TLI = 0.993, IFI = 0.999, and RMSEA = 0.075 (see Figure 2).

As shown in Figure 2, group affiliation and vocabulary are correlated, and both are associated with the social cognitive abilities of the children (as assessed by Hinting and CSCS). These social cognitive abilities, in turn, are linked to figurative language comprehension. Thus, social cognitive abilities serve as a mediating variable between group affiliation and vocabulary, influencing the children’s figurative language comprehension.

## 4. Discussion

This study’s findings suggest that the participants with TD scored higher in their understanding of idioms, irony, social cognition skills (understanding other’s intentions and social situations) compared to the participants with ASD matched according to age, gender, and nonverbal intelligence. These results reinforce the previous studies that compared figurative language and social competence between children with TD and children with ASD [3,12,31,33,34,35,45]. Consistent with our first hypothesis, these findings highlight a gap in pragmatics; there is reduced idiom and irony (ironic criticism) comprehension in the children with ASD compared to that of the children with TD aged 8–11-years-old matched according to chronological age, gender, and nonverbal intelligence, which is an age range in which these aspects of figurative are still developing.

Vocabulary seems to play an important role in shaping an understanding of figurative language and social situations, as the results of hierarchical regression analyses show. When the verbal and nonverbal intelligence scores were entered into the models, vocabulary (but not nonverbal intelligence) exhibited a significant unique contribution to idiom and irony comprehension. Specifically, 81% and 57% increases in EPV for idiom and irony comprehension, respectively, were observed. Thus, consistent with the claim that core language skills are closely related to figurative language comprehension in ASD [30] and the previous studies [12], our findings demonstrate that higher levels of vocabulary knowledge are associated with a better understanding of idioms and irony.

Our second hypothesis focused on the contributions of social cognitive skills (ToM and understanding social situations) to irony and idiom comprehension. The current results indicated that group affiliation, understanding social situations, and understanding the other’s intentions contributed to both the idiom and irony comprehension models. However, none of these variables were individually able to significantly predict idiom comprehension, despite the significant predictive performance of the overall step. In contrast, for irony comprehension, a significant unique contribution of ToM was observed for both the groups, but not of social situation understanding. In other words, being a child with TD, and thus enjoying a greater ability to understand others’ intentions, contributes to a better understanding of irony. This finding attests to the differential characteristics of the ironic stimuli, which involve understanding social scenarios in contrast to the idioms, which were provided without context. Our results contribute to the literature concerning the understanding of the link between children’s theories of mind and children’s developing social relationships. Children’s understanding of mental representations continues to grow in school age. The later developments include, among others, the interpretation of ambiguous events or moral dilemmas regarding truth and fairness and more subtle forms of social deception, such as white lies [41]. These later-developing ToM skills can lead to greater social harmony by reducing social misunderstandings. Thus, increased sensitivity to criticism (such as irony criticism) by school age potentially contributing to low self-esteem and anxiety [41]. Therefore, future studies should test the association between the developing ToM skills during school age, social understanding, and social relationships and pragmatics. Noteworthy are the nonsignificant interactions that were obtained in the fourth step, indicating that the effect of group affiliation on idiom or irony comprehension does not vary significantly, depending on the level of ToM or social situation understanding abilities.

The results of path analysis demonstrate that group affiliation and vocabulary are associated with social cognitive abilities (understanding other’s intentions and social situation understanding). These social cognitive skills, in turn, are linked to figurative language comprehension (idiom and irony comprehension). Thus, social cognitive skills serve as a mediating variable between group affiliation and vocabulary, influencing the ability to understand figurative language. As figurative language understanding lies in the interface between linguistics, cognition, and social cognition, our results indeed demonstrate a positive relationship between the social cognitive skills and the ability to understand idioms and irony. Figurative language, as an aspect of the broader domain of understanding language in a social context, namely pragmatics, is closely linked to ToM ability [5], and ToM ability is linked to an understanding of social situations [57]. To succeed in a social understanding task, the participants must encode the provided social information, interpret this information (such as cues), identify violations of behavioral norms (e.g., violating a teacher’s privacy by rummaging through the teacher’s bag), and understand the associated consequences, while also understanding the attitudes and intentions of each other. Consequently, there appear to be shared abilities underlying figurative language and social situation understanding. Our results indeed demonstrate that the better individuals’ understanding of social situations and others’ intensions are, the greater their understanding of figurative language (idioms and irony) is. Also note that vocabulary (but not group affiliation) has a direct association with figurative language comprehension.

There are several limitations to this study that should be noted. The first involves the difference in vocabulary knowledge between the groups. Although the children with ASD were high-functioning and recruited from communication classes in mainstream schools, they nonetheless demonstrated less vocabulary knowledge than the children with TD. Despite controlling vocabulary in our statistical analyses, these results are limited to children with TD matched to children with ASD by chronological age and nonverbal intelligence. A related limitation is that the participants’ grammatical skills were not evaluated. This means that children with potentially less-well-developed linguistic skills may have been disadvantaged in the linguistically demanding tests, both in terms of the comprehension of the texts and the test questions. Future studies should use, in addition to the vocabulary knowledge, a test assessing children’s grammatical skills. A further limitation of this study is the small sample size in both the groups of children, which restricts the generalizability of the findings. As a result, this research should be considered a pilot study, and it is recommended that future studies be conducted with a larger population for more robust conclusions. Another limitation of this study is the questionnaires that were used. Inspecting these questionnaires may also explain the differential results obtained for the idiom and irony models. For example, our findings show that vocabulary has a central role in the understanding of idioms and irony among children with and without ASD, beyond the effects of age and gender. Social abilities, including understanding the intentions of the other (ToM) and the ability to understand social situations, further increase their comprehension. However, ToM ability was found to uniquely contribute to irony, but not to idiom comprehension. This difference is likely attributable to the type of questionnaire used; the ironic stimuli were embedded within social situations, whereas the idioms were presented with no context. Future studies should consider using idioms embedded within social situations. Our results also indicated that ToM ability, but not performance on the social understanding questionnaire (CSCS), uniquely contributed to the understanding of irony. The CSCS, unlike the Hinting test, requires social knowledge to identify the violation of social norms, thus probably contributing less to participants’ performance when evaluating the ironic scenarios compared to the Hinting test. These findings strengthen the need to conduct future studies in more ecological settings that use various methods to assess social situation understanding (e.g., observations or interviews). A further limitation of this study is the omission of sociodemographic factors, such as the family environment and parental education. These variables could significantly influence the development of language competence in children, particularly those with autism spectrum disorder. Consequently, children classified at the same level of disorder, but raised in families with distinct sociocultural backgrounds may exhibit varying levels of language competence. We therefore recommend that future studies should balance the groups according to these sociodemographic factors.

## 5. Conclusions and Implications

In conclusion, vocabulary plays a major role in the understanding of idioms and irony among children with autism and their age-matched TD peers. Our findings also highlight the contributions of ToM ability and social situation understanding to the comprehension of both irony and idioms, although the ability to understand the intentions of others (ToM) uniquely predicted the understanding of irony (beyond vocabulary), but not idioms. Furthermore, the findings also show that group affiliation and ToM play a significant role in irony, but not in idiom understanding. Intervention programs aiming to enhance figurative language comprehension should consider using these social abilities to enhance the programs’ efficiency in promoting greater irony and idiom comprehension among children and adolescents with ASD and to examine the reciprocal effects of enhancing irony comprehension and the understanding of both social situations and others’ intentions, and vice versa. In other words, does enhancing irony comprehension improve social understanding skills? It would be also interesting to test whether ironic compliments and ironic criticisms differ in their associations with understanding social situations and understanding the intentions of others. Another potential implication of the current study is to explore the neural basis of understanding social situations, the intentions of others, and irony. It would be interesting to examine whether there is a neural overlap involved in these three abilities. Additionally, it would be intriguing to investigate whether it is possible to influence these abilities through electrical brain stimulation targeted at the neural network overlapping with these functions. This may improve the social skills of children with ASD.

## Figures and Tables

**Table 1:** Participant demographic and background characteristics.

	TD		ASD		Statistic	*p*
Gender, N (%)					X^2^ (1) = 0.35	0.553
Boys	17	(56.7%)	18	(64.3%)		
Girls	13	(43.3%)	10	(35.7%)		
Age	9.83	(0.65)	10.02	(0.52)	t (56) = 1.19	0.238
Vocabulary	58.30	(7.97)	43.07	(9.41)	t (56) = 6.67	<0.001
RAVEN	31.47	(3.46)	30.30	(4.86)	t (56) = 1.33	0.189

**Table 2:** A summary of the regression models for predicting the understanding of idioms and irony.

	Dependent Variables							
	Idioms Understanding				Irony Understanding			
Predictor Variables	B	SE	Beta	*p*	B	SE	Beta	*p*
Step I								
Gender	0.28	1.31	0.03	0.834	0.24	1.13	0.03	0.835
Age	−1.11	1.09	−0.14	0.316	−1.56	0.94	−0.22	0.104
$R^{2}$	0.019			0.597	0.048			0.262
Step II								
Nonverbal intelligence	0.12	0.08	0.11	0.111	−0.17	0.10	−0.17	0.085
Vocabulary	0.35	0.03	0.84	**<0.001**	0.31	0.04	0.83	**<0.001**
$\Delta R^{2}$	0.810			**<0.001**	0.573			**<0.001**
$R^{2}$	0.829			**<0.001**	0.620			**<0.001**
Step III								
Group affiliation	0.80	0.48	0.17	0.101	0.83	0.27	0.20	**0.003**
Social situation understanding	0.67	0.88	0.14	0.452	0.73	0.49	0.17	0.144
ToM	0.09	0.76	0.02	0.907	2.96	0.43	0.70	**<0.001**
$\Delta R^{2}$	0.028			**0.028**	0.321			**<0.001**
$R^{2}$	0.857			**<0.001**	0.942			**<0.001**
Step IV								
Social situation understanding	−0.28	0.79	−0.04	0.724	0.32	0.43	0.05	0.464
ToM	0.36	0.94	0.04	0.700	0.04	0.52	**0.01**	0.938
$\Delta R^{2}$	0.000			0.924	0.002			0.456
$R^{2}$	0.857			<0.001	0.944			**<0.001**

Note: Bold numbers indicate significant results.

## Data Availability

Data will be available on request due to privacy/ethical restrictions.
